# Supplementary material for: Divergent and Convergent TMEM106B Pathology in Murine Models of Neurodegeneration and Human Disease
Source: Res Sq. 2024 Nov 19:rs.3.rs-5306005. Preprint. [Version 1] doi: 10.21203/rs.3.rs-5306005/v1 (PMC11601866; doi:10.21203/rs.3.rs-5306005/v1)
Supplement: Supplement 1 [file NIHPPRS5306005V1-supplement-1.pdf]

Supplementary Files

This is a list of supplementary files associated with this preprint. Click to download.

- FigureS1.tiff
- FigureS2.tiff
- FigureS3.tiff
- FigureS5.tiff
- FigureS4.tiff
